# Determinants of cardiovascular disease and other non-communicable diseases in Central and Eastern Europe: Rationale and design of the HAPIEE study

**DOI:** 10.1186/1471-2458-6-255

**Published:** 2006-10-18

**Authors:** Anne Peasey, Martin Bobak, Ruzena Kubinova, Sofia Malyutina, Andrzej Pajak, Abdonas Tamosiunas, Hynek Pikhart, Amanda Nicholson, Michael Marmot

**Affiliations:** 1 International Institute for Health and Society, Department of Epidemiology and Public Health, University College London, UK; 2 Centre for Environmental Health, National Institute of Public Health, Prague, Czech Republic; 3 Institute of Internal Medicine, Russian Academy of Medical Sciences, Novosibirsk, Russia; 4 Department of Epidemiology and Population Studies, Institute of Public Health, Faculty of Health Care, Jagiellonian University Medical College, Krakow, Poland; 5 Department of Population Studies, Institute of Cardiology of Kaunas University of Medicine, Kaunas, Lithuania

## Abstract

**Background:**

Over the last five decades, a wide gap in mortality opened between western and eastern Europe; this gap increased further after the dramatic fluctuations in mortality in the former Soviet Union (FSU) in the 1990s. Recent rapid increases in mortality among lower socioeconomic groups in eastern Europe suggests that socioeconomic factors are powerful determinants of mortality in these populations but the more proximal factors linking the social conditions with health remain unclear. The HAPIEE (Health, Alcohol and Psychosocial factors In Eastern Europe) study is a prospective cohort study designed to investigate the effect of classical and non-conventional risk factors and social and psychosocial factors on cardiovascular and other non-communicable diseases in eastern Europe and the FSU. The main hypotheses of the HAPIEE study relate to the role of alcohol, nutrition and psychosocial factors.

**Methods and design:**

The HAPIEE study comprises four cohorts in Russia, Poland, the Czech Republic and Lithuania; each consists of a random sample of men and women aged 45–69 years old at baseline, stratified by gender and 5 year age groups, and selected from population registers. The total planned sample size is 36,500 individuals. Baseline information from the Czech Republic, Russia and Poland was collected in 2002–2005 and includes data on health, lifestyle, diet (food frequency), socioeconomic circumstances and psychosocial factors. A short examination included measurement of anthropometric parameters, blood pressure, lung function and cognitive function, and a fasting venous blood sample. Re-examination of the cohorts in 2006–2008 focuses on healthy ageing and economic well-being using face-to-face computer assisted personal interviews. Recruitment of the Lithuanian cohort is ongoing, with baseline and re-examination data being collected simultaneously. All cohorts are being followed up for mortality and non-fatal cardiovascular events.

**Discussion:**

The HAPIEE study will provide important new insights into social, behavioural and biological factors influencing mortality and cardiovascular risk in the region.

## Background

### The rationale for the study

The rationale for this research lies in the intriguing trends in cardiovascular (CVD) mortality seen in Europe over the past five decades. While in western Europe total and CVD mortality have been declining since the 1970s, in Central and Eastern Europe (CEE) and the former Soviet Union (FSU) rates have been increasing [1,2]. Dramatic fluctuations in mortality in Russia and other parts of the FSU in the late 1980s and the 1990s were superimposed on these long-term trends [3,4]. These trends have resulted in a large gap in life expectancy between eastern and western Europe [2,5].

Social factors act at the country (context) level and within countries. The population level trends provide the context, but it is important to understand how social conditions and lifestyle affect the health of individuals and groups within countries, rather than to examine trends. The rise of mortality in unmarried men [6,7], and the rapid increase in mortality in lower socioeconomic groups, resulting in dramatic widening of social inequalities within countries of CEE/FSU in recent years [8-14], suggest a powerful role of social factors. The question is how they affect cardiovascular disease in these societies which have undergone profound social change.

Analyses of the WHO MONICA data suggested that classical risk factors could explain only a part of the temporal changes and differences between populations [4,15-17]. Similarly, studies within populations, both in the east and the west of Europe, showed that only part of the socioeconomic differentials in CVD risk were explained by standard risk factors [18-21]. Other risk factors therefore need to be considered. Our main hypotheses relate to alcohol, nutrition and psychosocial factors. Binge and heavy drinking has been proposed as the explanation for the short-term fluctuations in mortality in Russia [3,22] but the evidence on the contribution of alcohol to trends in mortality and on the association between heavy and binge drinking and CVD in individuals remains inconclusive [23-25]. Nutrition, particularly low consumption of fruits and vegetables and its biomarkers (such as antioxidant vitamins or folate), has been linked with CVD risk [26,27] and has been shown to be low in CEE/FSU [28-31]; however, direct individual-based evidence on its role in the high mortality in the region is insufficient. The link between material hardship and psychosocial distress is well documented [32] but, again, direct evidence on their independent effects on CVD in CEE/FSU is sparse. There is important circumstantial evidence supporting each of these hypotheses; the ultimate scientific test, however, requires longitudinal studies of individuals.

## Methods and Design

### Hypotheses and research questions

To investigate the determinants of cardiovascular diseases and other chronic conditions in Central and Eastern Europe, we are conducting a prospective cohort study in Russia, Poland, the Czech Republic and Lithuania. The study will investigate the following specific hypotheses:

• Socioeconomic factors are key determinants of health in CEE/FSU; we will examine the pathways involved in their action, including factors hypothesised below.

• Psychosocial factors, both at individual and population level, are related to CVD and other non-communicable diseases.

• Low consumption of fresh fruits and vegetables and their nutrient biomarkers are associated with increased risk of CVD;

• Binge drinking and heavy alcohol consumption are related to all-cause mortality, CVD and injury;

• Elevated concentration of homocysteine and low levels of folate and related B vitamins are associated with increased risk of CVD;

• Interactions between different groups of risk factors, in particular between heavy drinking and folate deficiency, and between the MTHFR genotype and folate deficiency, are associated with CVD.

In addition to these specific hypotheses, the study will also investigate several more general questions:

• The role of childhood socioeconomic circumstances and biological markers of their effects, such as leg length and lung functions, in the risk of CVD and other conditions in adulthood;

• Biological, social, economic and psychosocial determinants of healthy ageing (cognitive function, physical functioning, and quality of life of elderly persons);

• Genetic predictors and non-conventional biomarkers of CVD and other chronic diseases.

### Study populations and subjects

The HAPIEE (**H**ealth, **A**lcohol and **P**sychosocial factors **I**n **E**astern **E**urope) study consists of three cohorts in Novosibirsk (Russia), Krakow (Poland) and six centres in the Czech Republic (Havirov/Karvina, Hradec Kralove, Jihlava, Kromeriz, Liberec and Usti nad Labem). A fourth cohort is currently being established in Kaunas, Lithuania.

The cohorts consist of random samples of men and women aged 45–69 years old at baseline, stratified by gender and 5 year age groups, and selected from population registers. The planned sample size was 10,000 persons in each country; the actual study size 28,947 individuals (Table 1). In Russia, both the questionnaire and the examination have been completed in a clinic. In Poland and the Czech Republic, the subjects were first visited at home, to complete a structured questionnaire, and then invited to a clinic for a short examination. For this reason, not all subjects have data on both questionnaire and examination; the proportion of subjects with full data is 82% in the Czech Republic and 87% in Poland. Lithuania joined the project in 2005; the planned size of the Lithuanian cohort is 7,000 men and women aged 45–69 years, randomly selected from the population register of the city of Kaunas. The study was approved by the ethics committee at University College London, UK and by the ethics committee in each participating centre. All participants gave written informed consent.

### Baseline data collection

The baseline survey in Russia, Poland and the Czech Republic was conducted in 2002–2005; data were collected by structured questionnaires and examination in clinic including a fasting venous blood sample. The questionnaire covered *health *(self-rated health status, medical history, health behaviours, physical functioning (from the SF36 instrument)[33,34]; *life style*, *food frequency *(in last 3 months), *socioeconomic circumstances *(own and parental education; economic status; type of employment; ownership of car and other household items); *psychosocial factors *(perceived control [35], the 20 item CESD scale of depression [36]); quality of life of retired persons (the CASP19 questionnaire) [37]; and *psychosocial environment at work *(job demand and job control [38] and effort-reward imbalance [39,40]). All questions were translated from English into each language and back translated into English to check for accuracy.

The short examination included measurement of height, weight, trunk length, waist and hip circumference, blood pressure, lung function and cognitive function (memory, concentration and verbal skills). Prior to blood pressure measurement participants were asked to sit quietly for 5 minutes. Blood pressure was measured three times, with a two minute interval between measurements, using an Omron M5-I digital blood pressure monitor. Lung function was assessed with a Micro-Medical Microplus spirometer (using Spida 4 software to store curves in electronic format). Forced vital capacity (FVC), peak expiratory volume in 1 second (PEV1), and peak expiratory flow (PEF) were recorded. Cognitive function tests involved three immediate and one delayed recall of 10 words, animal naming in 1 minute, and letter cancellation in 1 minute.

Blood samples were collected in Becton Dickinson SST II (10 ml) and K_2_-EDTA vacutainers (10 ml and 2 × 3 ml). All vacutainers were stored at 4 degrees Celsius prior to processing. The 10 ml SST II and 10 ml K_2_-EDTA vacutainers were centrifuged at 4000 rpm for 15 minutes, and serum and plasma samples were divided into 4 and 3 aliquots respectively. In addition, one 250 μl aliquot of plasma was stabilised with 250 μl of 10% metaphosphoric acid for subsequent vitamin C determination. The two 3 ml K_2_-EDTA vacutainers were not centrifuged; one vacutainer (destined for glycated haemoglobin determination) was divided into 2 aliquots × 1.5 ml. All aliquots were stored in 1.5 ml Sarstedt microtubes, and together with remaining 3 ml K_2_-EDTA vacutainer (for DNA extraction) were stored at -80°C for subsequent laboratory analysis. DNA has now been extracted, divided into 3 aliquots and stored at -20°C.

Data entry of baseline questionnaires and medical examination data in the Czech Republic and Russia was done using Epi-Info 6 software (CDC, Atlanta, USA), and questionnaires were electronically scanned in Poland. A proportion of forms were double entered for quality assurance. Baseline questionnaires in Lithuania are being completed using CAPI (see re-examination section for details) and medical examination data is double entered in Epi-Info 6.

Table 2 summarises the data available from the baseline survey. All data from questionnaire and examination are now available. Total cholesterol, HDL cholesterol and triglycerides have been measured in the Czech Republic, Poland and Russia. Central laboratory analyses are in progress for a random sub-sample of 1,000 participants per country (including determination of folate, vitamin B12, homocysteine, glycated haemoglobin, vitamin C, alfa-tocopherol, beta-carotene, retinol and C-reactive protein). Baseline examination of the Lithuanian cohort began in Spring 2006 and is expected to be completed by summer 2008.

### Re-examination of the cohorts

Re-examination of the cohorts started in spring 2006 and is planned to take 2 years in all four countries. The main focus of the re-examination is healthy ageing and economic well-being. The data are being collected by face-to-face Computer Assisted Personal Interview (CAPI) using Blaise 4.6 software (Statistics Netherlands). The generic version of the CAPI program is in English, but all questions have been forward and backward translated into each language to ensure consistency of questions in all countries.

The *ageing-related *outcomes include: cognitive functions (as in baseline); physical functioning (12 items from SF-36 questionnaire [33,34]; activities of daily living (ADL), instrumental activities of daily living (IADL), walk speed, chair rise and grip strength; quality of life (12-item CASP questionnaire[37,41] and social participation. The walk speed test records the time to walk a distance of 2 m at usual speed, while the chair rise test records the time to stand up and sit down 5 times without using their arms. A Scandidact dynamometer is used to measure the maximum grip strength of each hand.

The *economics *measures include details on retirement; household composition; formal and informal household income; household wealth; and expectations of future pensions. Table 2 summarises the data being collected during re-examination of the cohort. In Lithuania, baseline data collection and collection of additional data on healthy ageing and economic well-being will be collected simultaneously. The re-examination has been designed in such a way that the data on ageing and economics will be directly comparable with the English Longitudinal Study of Ageing [42], the Study of Healthy Ageing and Retirement in Europe (SHARE) [43], and Health and Retirement Study (HRS) in the USA [44].

### Follow up of the cohorts

The study has two primary outcomes of interest: a) mortality from all causes and from CVD, and b) non-fatal cardiovascular events. Over the last year, the follow-up mechanisms for these outcomes have been piloted.

#### Mortality

We use the following data sources on mortality. In Novosibirsk, we use the death register developed by the Institute of Internal Medicine. The register is based on data from medical death certificates, the Novosibirsk office of the State Statistical Bureau (Goscomstat) and from the population registration bureau (ZAGS). The system has been in place for a number of years and provides complete coverage of deaths in the study population [18,24]. In Krakow, we use the provincial death register covering the city of Krakow and surrounding area. In the Czech Republic, we use the national death register. In Lithuania, we use the Kaunas regional mortality register. In the past, the register has been shown to provide a complete coverage of deaths in Kaunas [45].

#### Non-fatal cardiovascular events

The availability of data on non-fatal cardiovascular events differs by country. In Russia and in Lithuania, there are existing registers of myocardial infarction (MI) and stroke established by the WHO MONICA Project [46], but not in Poland and the Czech Republic. In order to obtain comparable data in all four countries, we piloted the mechanisms to (1) identify, (2) confirm and (3) validate incident non-fatal cardiovascular events.

##### (1) Identification

Postal questionnaire is the primary source for identifying cases in all 4 countries. The questionnaire was sent out in spring 2005 and 2006, will be repeated during the cohort re-examination in 2006–2008 and will then be sent by post every 3 years. Potential CHD cases are identified by positive responses to questions on history of MI, cardiac procedures or history of stroke. To identify events for non-responders to the postal questionnaire and re-examination phase, we will use the MI and stroke registers in Russia and Lithuania, and hospital databases in the Czech Republic (and hopefully in Poland). The proportion of MI cases identified by questionnaire and by these other sources will be compared in order to estimate the potential loss of cases in countries without registers.

##### (2) Confirmation

Potential incident cardiovascular events (MI, unstable angina and stroke, or diagnostic or therapeutic procedures such as angioplasty or CABG), identified from data sources described above, will be validated using the following information: discharge reports and hospital/medical records. If the discharge report indicates a potentially acute incident coronary event, further information on signs, symptoms and enzymes, and copies of ECGs are sought from hospital records, to enable validation of hospital diagnosis and application of different MI criteria. Prevalent cardiovascular events will not undergo full validation but we plan to obtain hospital discharge data wherever possible to increase the accuracy of the baseline data. For stroke cases, discharge and/or hospital reports are collected on each episode of stroke identified by the postal questionnaire and by stroke registers in Russia and Lithuania, to provide the rates of clinically confirmed strokes, and allow comparison between countries.

##### (3) Validation

We will use a simplified version of the criteria developed by the American Heart Association [47]. We are collecting an extensive range of data on each event, including signs, symptoms, biomarkers (including non-troponin enzymes) and, where possible, at least 3 copies of ECG. This will allow us to apply different sets of criteria, including those less stringent than AHA, such as the MONICA criteria [46]. Stenosis of more than 70% in any artery or 50% in left main artery, PCI, and CABG are considered as indicating CHD.

### Statistical analysis

On the basis of the follow up conducted so far, we estimate that by the end of 2008 and 2010, i.e. on average 4 and 6 years after the baseline survey, there will be a total of about 1360 and 2180 deaths, respectively, from all causes across the three existing cohorts (without Lithuania), approximately half of them from CVD. In terms of non-fatal events, we estimate that by the end of 2009 (postal CVD questionnaire will be repeated in spring 2010) there will be approximately 750 new coronary events and approximately 350 new strokes. These numbers will provide the study with sufficient statistical power to analyse the relationship between proposed risk factors and mortality and non-fatal events and thus to test the research hypotheses.

Baseline data are currently being analysed, but the main hypotheses will be tested using the longitudinal data. After initial exploratory analyses, age-adjusted effects will be estimated by Cox proportional hazard modelling, separately for each country and separately for men and women. These estimates will provide the first estimates of the effects of the independent factors and will show whether there could be effect modification by (heterogeneity between) sex and country. Possible clustering of subjects within cities will be taken into account. Multivariate Cox regression models will be used to estimate independent effects of the suspected risk factors, taking into account potential confounders and effect modifications. Possible pathways linking risk factors with the outcome will be set out a priori, and associations and pathways will be confirmed by structural equation modelling. In addition, data from baseline and re-examination will be used to assess changes in risk factors over time (e.g. smoking, heavy drinking) and intermediate health characteristics (presence of diabetes, hypertension or angina).

The analyses will focus on data from the HAPIEE cohorts but in some instances, comparisons with western populations will be important. There are several such comparison groups. First, we have access to data from a small cohort (n = 1007, the same age groups as HAPIEE cohorts) set up in 2003 in southern Sweden under direction of Dr Margareta Kristenson, University of Linkoping, using a similar protocol and many identical measurements. Second, we will compare psychosocial determinants of morbidity and mortality with the Heinz Nixdorf-RECALL study [48]. Third, we will use data from Whitehall II study of civil servants [49] with identical measurements of many lifestyle and psychosocial factors. Fourth, we have access to data from the English Longitudinal Study of Ageing [42], housed in the UCL Department of Epidemiology and Public Health, with measurements of ageing related outcomes and economics identical to those in the HAPIEE study.

## Discussion

The HAPIEE study will provide valuable insight into the determinants of CVD and other chronic conditions in Eastern Europe and the former Soviet Union where health and mortality have worsened dramatically over a very short period, coinciding with transition from communism to market economy. Such societal transition is often accompanied by a rapid increase in social inequalities and social distress. Currently the understanding of trends in, and determinants of, health in such societies is patchy at best. The results from HAPIEE study will be relevant for policies aiming to alleviate the impact of transition in other populations.

This large cohort study with extensive measurements and a rich bank of biological and genetic samples will be used to test both existing and new hypotheses concerning causes of disease and ill health to confirm that associations between risk factors and disease seen in the west can be replicated in non-western populations. It will investigate the social patterning of health and, because the social structure differs from the west, will increase understanding of the links between social environment and health. The current focus on healthy ageing is important, since age-related onset of disability in Russia seems faster than in the west [50,51] but so far little is known about the determinants of health among the elderly in Eastern Europe.

The most serious challenge in this study was to achieve satisfactory response rates. Our projection of the study size of 30,000 persons was based on response rates in earlier studies in Eastern Europe in the late 1990s but response rates in the region have since declined rapidly. Although our final response rates are typical for current studies in Eastern Europe, and elsewhere [52-56], careful assessment of the non-response bias is nevertheless important. In all centres, we collected short questionnaires from sub-sample of those who refused participation. In Novosibirsk and Krakow, we conducted extra home visits in a sub-sample of non-respondents; in the Czech Republic we assessed the completeness of the population registers in the Czech town with the largest number of invitations. The analysis of these data and the comparison of participants and non-respondents showed two important features.

First, a non-negligible proportion of non-respondents had moved away or died before the start of the study and were therefore not eligible for the study. Extrapolating from the proportion of incorrect addresses identified in home visits in Krakow and Novosibirsk and from the assessment of the accuracy of the population register, the real response rates are higher than those shown in Table 1 – at least 68% in Krakow, at least 71% in Novosibirsk and over 60% in the Czech Republic. A further proportion of non-respondents could not be contacted after 3 home visits, and many of these may not live at their officially registered address. The true response rates may therefore be even higher.

Second, participation rates were higher in women, increased with age, and participants had higher education, better self-rated health and lower prevalence of smoking than non-respondents. This confirms the general experience that responders in epidemiological studies are healthier than non-responders [57]. Response rates and healthy volunteer bias may complicate comparisons between cohorts but they will not have a large influence on within cohort analyses.

The HAPIEE study is one of the largest prospective studies of non-communicable diseases ever conducted in Eastern Europe, and it is one of the first that will systematically investigate healthy ageing and its determinants. The results of the study will provide important insights into social, behavioural and biological risk factors of cardiovascular diseases, injuries, depression and other conditions common in the region.

## Competing interests

The author(s) declare that they have no competing interests.

## Authors' contributions

A. Peasey participated in the design and coordination of the study, and drafted the manuscript. MB, HP and MM participated in the design and coordination of the study and helped draft the manuscript. RK, SM, A. Pajak, AT and AN participated in the coordination of the study and helped draft the manuscript. All authors read and approved the final manuscript.

## Pre-publication history

The pre-publication history for this paper can be accessed here:



## Figures and Tables

**Table 1:** Absolute numbers and response rates in HAPIEE study

	***Men***	***Women***	***Total***	***Response rate (%)***
Russia	4,269	5,094	9,363	61%
Poland	5,230	5,498	10,728	61%
Czech Rep.	4,125	4,731	8,856	55%

Total	13,624	15,323	28,947	59%

**Table 2:** Overview of questionnaire and medical examination data available in the HAPIEE study

**Domain**	**Variables**	**Wave I (Baseline)**	**Wave II**
Health	Medical history	Y	Y
	Current medication	Y	Y
	Self-rated health	Y	Y
	Alcohol, pattern & problem drinking	Y	Y
	Awareness & treatment of high BP, high cholesterol and diabetes	Y	Y
	Depression (CESD-20)	Y	-
	Depression (CESD-10)	-	Y
	Injuries in last year	Y	Y
	Food frequency (fruit & vegetable intake, seasonality)	Y	-
	Expectations of future health and length of life	-	Y
	Anthropometry (height, weight, waist and hip circumference, and sitting height)	Y	-
	Blood pressure	Y	-
	Heart rate	Y	-
	Pulmonary functions (FVC, FEV1 and PEF)	Y	-
	Blood sample	Y	-

Physical functioning	Physical functioning (from SF36)	Y	Y
	ADL & IADL	-	Y
	Walk speed	-	Y
	Chair rise	-	Y
	Grip strength	-	Y

Cognitive functions	Memory (immediate and delayed word recall)	Y	Y
	Concentration	Y	Y
	Speed	Y	Y
	Numeracy	-	Y (LT only)

Social status	Education	Y	Y
	Marital status	Y	Y
	Childhood social circumstances	Y	Y
	Brief employment history	Y	Y
	Self-assessed material deprivation	Y	Y
	Life-grid of past social status	-	Y
	Housing conditions/crowding	-	Y

Psychosocial factors	Social networks	Y	Y
	Perceived control over life	Y	Y
	Job control/demand (working only)	Y	-
	Effect/reward imbalance (working only)	Y	Y
	Social participation	Y	Y
	Happiness	-	Y

Economics	Household amenities	Y	Y
	Current economic activity	Y	Y
	Current labour market status	Y	Y
	Personal income	-	Y
	Household income and wealth	-	Y
	Housing tenure	-	Y
	Benefits	-	Y
	Informal transfers	-	Y
	Non-monetary income	-	Y
	Self-production	-	Y
	Expectations of future economic position	-	Y

Retirement	Type of retirement	-	Y
	Reasons for retirement	-	Y

Quality of life	CASP-19 (retired only)	Y	-
	CASP-12 (all participants)	-	Y

Community-level	Social trust	Y	Y
characteristics	Perception of reciprocity	-	Y
	Membership in civil organizations	-	Y
	Collective efficacy	-	Y
	Area characteristics from official sources	-	Y

Legend Y = yes; CESD = Center for Epidemiological Studies – Depression scale ; FVC = forced vital capacity; FEV1 = forced expiratory volume in 1 second; PEF = peak expiratory flow; ADL = activities of daily living; IADL = instrumental activities of daily living; LT = Lithuania; CASP = Control, Autonomy, Self-realization and Pleasure.
